# Piezoelectric enhanced sulfur doped graphdiyne nanozymes for synergistic ferroptosis–apoptosis anticancer therapy

**DOI:** 10.1186/s12951-023-02059-y

**Published:** 2023-09-02

**Authors:** Jianxin Wang, Yinzhu Chu, Zhiyu Zhao, Cong Zhang, Qi Chen, Haitao Ran, Yang Cao, Changjun Wu

**Affiliations:** 1 Department of Ultrasound, The First Affiliated Hospital of Harbin Medical University, Harbin, 150001 China; 2 Chongqing Key Laboratory of Ultrasound Molecular Imaging, Institute of Ultrasound Imaging, Second Affiliated Hospital, State Key Laboratory of Ultrasound in Medicine and Engineering,, Chongqing Medical University, Chongqing, 400010 China

**Keywords:** Graphdiyne, Nanozymes, Ferroptosis, Ultrasound, Piezoelectric catalytic therapy

## Abstract

Graphdiyne has excellent potential due to its enzymatic properties. Metal-free sulfur-doped Graphdiyne (S-GDY) has piezoelectric characteristics, and ultrasonic excitation of S-GDY enhances peroxidase activity. It can turn hydrogen peroxide into toxic hydroxyl radicals and induce apoptosis in 4T1 cells. More importantly, the ultrasound (US) enhanced nanozyme induced 4T1 cell ferroptosis by promoting an imbalanced redox reaction due to glutathione depletion and glutathione peroxidase 4 inactivation. S-GDY exhibited enhanced nanozyme activity in vitro and in vivo that may directly trigger apoptosis-ferroptosis for effective tumor therapy. Altogether, this study was expected to provide new insights into the design of piezoelectric catalytic nanozyme and expand their application in the catalytic therapy of tumors.

**Graphical Abstract:**

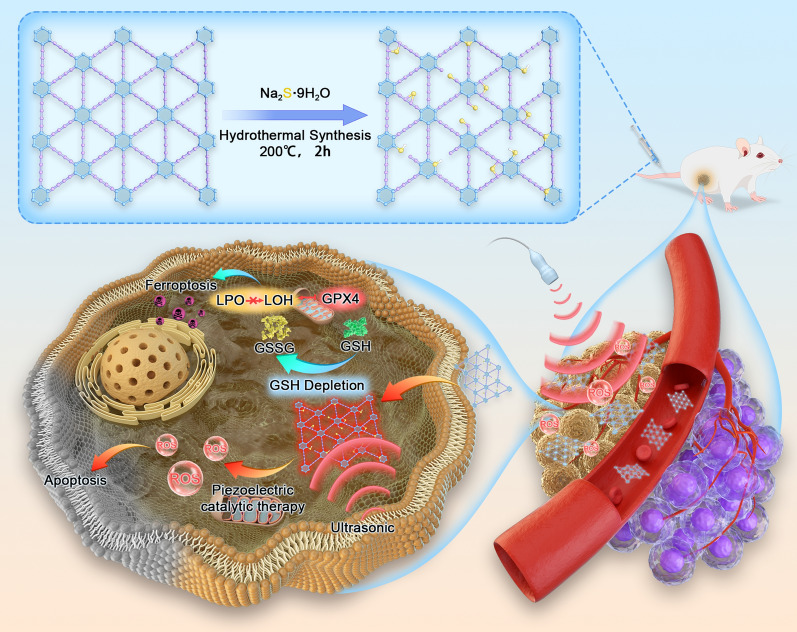

**Supplementary Information:**

The online version contains supplementary material available at 10.1186/s12951-023-02059-y.

## Introduction

Nanozymes, as artificial enzymes of nanomaterials with enzyme-mimicking activity [1–3]. These have higher physicochemical stability, higher durability, and lower cost in harsh environments compared with natural enzymes, which have attracted the extensive attention of researchers [4–6]. A large number of nanomaterials have been studied and confirmed to have mimetic activities of enzymes, such as peroxidase (POD), catalase, and superoxide dismutase [7–9]. Nanozymes mainly comprise metals and metal oxides because metal active sites can effectively mimic the redox process catalyzed by natural enzymes [10, 11]. However, introducing metals into in vivo treatment will inevitably cause unavoidable tissue damage and toxicity, limiting its clinical transformation and application [10]. Therefore, exploring safer and more innovative nanozymes for biomedical applications is necessary.

Graphdiyne (GDY), a recently discovered member of carbon-based nanozymes, is an ideal candidate for disease diagnosis and treatment due to its catalytic activity, excellent biocompatibility, and non-toxicity [11–14]. GDY comprises a central benzene ring and a butadiene linker, contains both sp and sp^2^ hybridized carbon atoms, and has the same hexagonal symmetric structure as graphene [15, 16]. The sp and sp^2^ hybridized carbon atoms in GDY make the surface charge distribution uneven and make GDY have high intrinsic activity, which provides favorable conditions for the preparation of highly active metal-free electrocatalysts [17–20]. Research showed that GDY has POD-like activity, which can catalyze hydrogen peroxide (H_2_O_2_) in the tumor microenvironment to generate toxic reactive oxygen species (ROS), especially hydroxyl radicals(·OH,21). Doping with non-metal atoms can form more active sites and defects, which is another effective way to improve the catalytic performance of GDY [21, 22].

Introducing external stimuli (such as ultrasound, light, heat, etc.) into the reaction system catalyzed by nanozyme can enhance the enzymatic activity without changing the intrinsic properties of nanozyme [23, 24]. Ultrasound has the characteristics of high tissue penetration and small energy attenuation [23, 25, 26]. The cavitation effect can produce bubbles that release strong local stress when ruptured. Once subjected to the stress the cavitation gas rupture releases, the piezoelectric material can immediately generate a built-in electric field and a surface piezoelectric potential [23]. The formed surface piezoelectric potential can transfer mechanical energy, which allows charge carriers to pass through the piezoelectric material surface or solution interface and trigger variable redox processes [23, 27–29]. Therefore, heteroatom doping and ultrasound can be combined to increase the activity of GDY nanozymes, thereby increasing the yield of •OH and inducing more tumor cell apoptosis.

Ferroptosis is an iron-dependent cell death mode significantly different from apoptosis, and the combined strategy of ferroptosis and apoptosis may bypass the inhibition of apoptosis to obtain a better therapeutic effect [30–32]. In short, ferroptosis is mainly regulated by the glutathione (GSH) and glutathione peroxidase 4 (GPX4) redox system. GSH depletion led to the inactivation of GPX4; the GPX4-catalyzed glutathione reductase reaction cannot metabolize lipid oxides, and lipid peroxidation (LPO) accumulation induces ferroptosis [33–37]. Therefore, the development of apoptosis-ferroptosis as a promising tumor therapy is highly necessary.

**Scheme 1.**
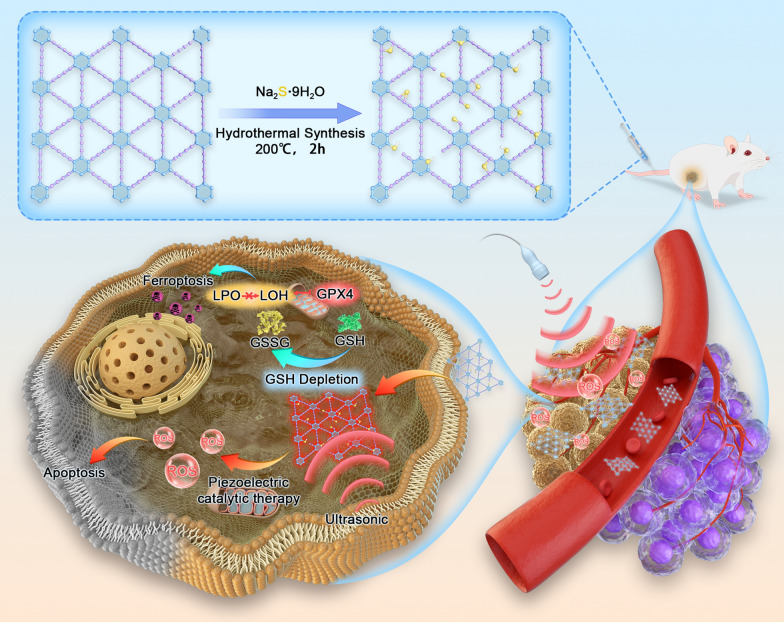
Schematic illustration of the synthesis of S-GDY nanozymes and the mechanism of piezoelectric catalytic enhancement of tumor synergistic apoptosis–ferroptosis therapy

Herein, we used GDY and sodium sulfide (Na_2_S) to synthesize metal-free S-GDY nanosheets (Scheme 1). S-GDY was a highly efficient peroxidase mimic and exhibits nonparallel piezoelectric responsiveness for enhanced enzyme mimicry activity. More importantly, ultrasound-enhanced nanozymes can induce 4T1 cells to ferroptosis by inducing GSH depletion and GPX4 inactivation and breaking the balance of redox. S-GDY exhibited enhanced enzyme activity in vitro and in vivo that may directly trigger apoptosis-ferroptosis for effective tumor therapy. Altogether, this study was expected to provide innovative insights into the design of piezoelectric catalytic enzymes and expand their application in the catalytic therapy of tumors.

## Experimental

### Materials and reagents

H_2_O_2_ (30 wt%), and GSH were purchased from Aladdin Chemical Reagent Co., Ltd. Sodium sulfide nonahydrate, tetrahydrofuran, and pyridine were purchased from Sinopharm Chemical Reagent Co., Ltd. 5,5′-dithio-bis (2-nitrobenzoic acid) (DTNB), and 3,3′,5,5′-Tetramethylbenzidine (TMB) were bought from J&K Chemical Co., Ltd.2′-7′-dichlorofluorescein diacetate (DCFH-DA) was purchased from Sigma-Aldrich. Anti-Caspase-3 and anti-GPX4 antibodies were purchased from Abcam. Cell counting kit-8 (CCK-8) assay and Liperfluo probe were purchased from tongren institute of chemistry. DiR fluorescent dye was purchased from MedChemExpress. DiI fluorescent dye and 4′,6-diamidino-2-phenylindole (DAPI) were obtained from Beyotime Biotechnology.

### Instruments

Characterization of S-GDY nanosheets using transmission electron microscopy (TEM, 2000 FX, JEOL, Japan). Piezoelectric response force microscopy (PFM) was performed on an atomic force microscope (Bruker Dimension Icon, Germany). The elemental analysis of S-GDY was determined by an X-ray photoelectron spectrometer (XPS, Thermo Fisher Scientific, USA).

### Preparation of S-GDY

GDY powder (15 mg) was dispersed in ultrapure water (15 mL) and irradiated with an ultrasonic wave for 30 min. Then the prepared solution and Na_2_S solution are mixed evenly and transferred to a stainless steel autoclave lined with polytetrafluoroethylene, conducting a hydrothermal reaction at 200 ℃ for 2 h. The obtained nanosheets were collected through centrifugation and washed several times with ethanol and ultrapure water. The final nanosheets were freeze-dried for future use.

### The POD-like activity of S-GDY and density functional theory (DFT) calculations

The POD-like activity of S-GDY was measured by measuring oxidized TMB's absorbance at 652 nm using a UV–Vis spectrophotometer (Cary 5000, Agilent Technologies Inc., USA). Briefly, S-GDY or GDY dispersions (100 μg mL^−1^) were added to 3 mL of NaAc buffer (pH = 4.0) containing TMB (1 mM) and H_2_O_2_ (50 mM). The solutions were treated with or without ultrasonic radiation (1 W/cm^−2^). TMB and H_2_O_2_ were added to the Control and Control+ US groups, without adding GDY or S-GDY. The absorbance was measured as a function of reaction time. DFT calculations were performed as described in detail in the literature [21].

### GSH depletion by S-GDY

S-GDY (50 μg mL^−1^) and H_2_O_2_ (50 μM) were mixed with GSH (2.5 mM) in Tris–HCl buffer (1 mL) at room temperature. The control groups were prepared by replacing S-GDY+ H_2_O_2_ with S-GDY, H_2_O_2_, or ultrapure water. At different time points, the above mixture and DTNB were added sequentially into Tris–HCl buffer (1 mL). A microplate reader quantified the amount of GSH remaining in the mixed solution by measuring absorbance at 412 nm.

### Cellular uptake

4T1 cells were seeded in confocal laser scanning microscopy (CLSM) dishes cultured at 37 ℃ in 5% CO_2_ for 24 h. After removing the culture medium, S-GDY nanosheets labeled with DiI were added and incubated with 4T1 cells at different times. The nanosheets were washed with phosphate buffered saline (PBS), and the nuclei were labeled with DAPI. The cellular uptake ability of S-GDY was analyzed using CLSM.

### Evaluation of ROS production in 4T1 cells

4T1 cells were seeded in CLSM dishes cultured at 37 ℃ in 5% CO_2_ for 24 h to detect total ROS production. CLSM dishes were randomly distributed into four groups: Control, ultrasound, S-GDY, and S-GDY+ US. We treated 4T1 cells according to the above groups and stained each group with DCFH-DA (10 μM) to observe the production of ROS using CLSM. The ROS production was analyzed by flow cytometry according to the manufacturer’s instructions.

### Intracellular GSH detection

4T1 cells were seeded in 12-well plates and randomly distributed into four groups: Control, US, S-GDY, and S-GDY + US. After corresponding treatments, the GSH content in each well was quantified with a GSH assay kit following the manufacturer’s protocol.

### Western blot analysis

The above four groups of proteins were extracted, and then electrophoresis, transfer, and blocking were performed. Protein is incubated with anti-Caspase-3, anti-GPX4, and anti-β-actin antibodies overnight at 4 ℃. The rabbit IgG second antibody conjugated with horseradish peroxidase was incubated in a shaker at room temperature for 1 h. The protein bands were then developed.

### Evaluation of LPO accumulation in 4T1 cells

4T1 cells were seeded in CLSM dishes cultured at 37 ℃ in 5% CO_2_ for 24 h. 4T1 cells were seeded in CLSM dishes and incubated overnight. CLSM dishes were randomly distributed into four groups: Control, ultrasound, S-GDY, and S-GDY+ US. Use Liperfluo (1 μM, 20 min) probe to stain LPO and visualize it using CLSM.

### Cell viability study and apoptosis assay

4T1 cells were seeded into a 96-well plate at a density of 1 × 10^4^ cells per well. After cultured at 37 ℃ with 5% CO_2_ overnight, divide wells into the following groups: Control, US, S-GDY, and S-GDY+ US. Then, the viability of 4T1 cells was measured by a CCK-8 assay according to the manufacturer’s instructions. 4T1 cells were seeded into a 6-well plate and incubated overnight for cell apoptosis assay. After corresponding treatments, the cell apoptosis rates were detected using a flow cytometer.

### The pharmacokinetics and metabolism of S-GDY in vivo

The Animal Experiment Center of Chongqing Medical University approved all animal experiment procedures. The tumor model was established by subcutaneously injecting 4T1 cells (cell count 5 × 10^5^). When the tumor volume reached about 70 − 80 mm^3^, subsequent animal experiments will be conducted. Inject DiR-labeled S-GDY nanosheets (DiR: 0.5 mg kg^–1^) into mice through the tail vein, and at different time points after injection, collect mouse blood from the orbit and centrifuge to obtain serum. Quantitative fluorescence (FL) intensity using a fluorescence imaging system. Collect data for fitting. In addition, 4T1 tumor-bearing mice were injected with DiR-labeled S-GDY nanosheets to observe the biological distribution and tumor-targeting ability of S-GDY in vivo.

### Anti-tumor therapy efficacy in vivo

4T1 tumor-bearing mice were randomly divided into four groups: Control, US, S-GDY, and S-GDY+ US. After intravenous injection of S-GDY (3 mg mL^−1^,200 μL), 2.0 W cm ^−2^ US (on for 3 min, off for 3 min, four cycles) was applied to irradiate the tumor area. The same treatment was repeated 24 h later. During the treatment period, the tumor volume and weight of the mice were recorded every 2 days. After 14 days, the tumors of each group of mice were weighed and photographed. Take the treated tumors from each group for TdT-dependent dUTP-biotin nick end labeling (TUNEL) and hematoxylin and eosin (H&E) staining, and collect the main organs for H&E staining. GPX4 in tumors was detected through Western blot assay. GSH in tumors was also detected using a GSH assay kit. The remaining mice were used to observe the survival rate of each treatment group.

### Biological safety of S-GDY nanosheets

Inject S-GDY nanosheets (3 mg mL^−1^,200 μL) intravenously into healthy mice to evaluate their biological safety. At a specific time point (1, 7, and 14 d), mice were sacrificed, and blood was collected for blood routine and biochemical tests. Collect the main organs for H&E staining.

### Statistical analysis

Quantitative data are presented as mean ± SD. Unpaired two-tailed Students’ *t*-test determined statistical comparisons between two groups. One-way analysis of variance (ANOVA) was used to compare the difference among multiple groups. The Kaplan–Meier method and the log-rank test were used to estimate the survival rates. All experiments were repeated at least three times. The *P*-value less than 0.05 was considered significant.

## Results and discussion

### Synthesis and characterization of S-GDY nanosheets

**Fig. 1:**
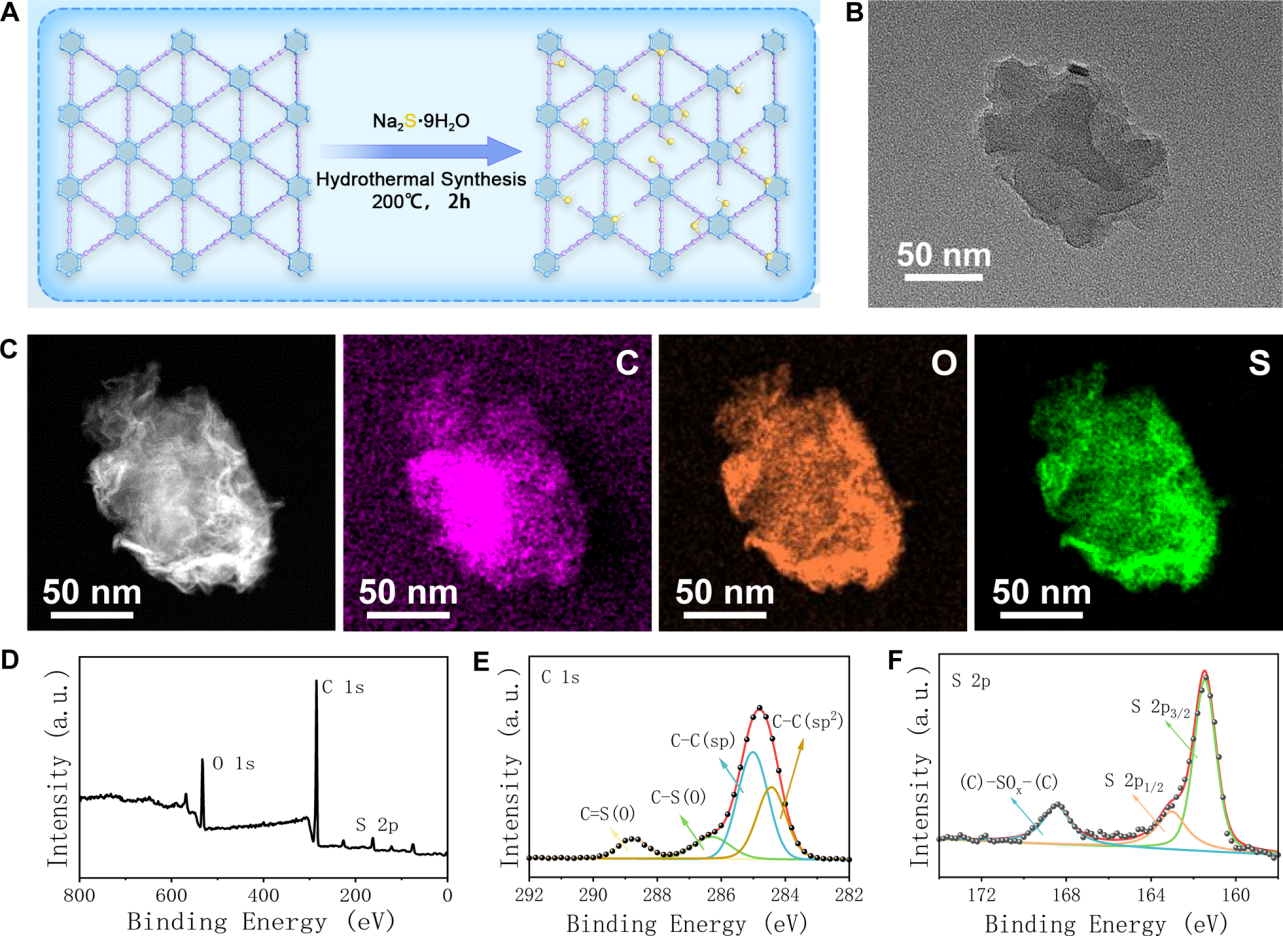
Synthesis and characterization of S-GDY. **A** The synthesis of S-GDY, **B** TEM image of S-GDY, **C** High resolution TEM and element mapping images of S-GDY, **D** XPS of S-GDY, **E** XPS C 1 s spectra of S-GDY, **F** XPS S 2p spectra of S-GDY

S-GDY nanosheets were synthesized by a hydrothermal approach using GDY and sodium sulfide nonahydrate (Fig. 1A). The resultant S-GDY was characterized by TEM (Fig. 1B), and elemental mapping images show that carbon, oxygen, and sulfur elements are uniformly distributed in the S-GDY nanosheets (Fig. 1C). The oxygen element may originate from the absorbed oxygen introduced during the synthesis [21]. The XPS spectrum was used for the elemental composition of the S-GDY nanosheet (Fig. 1D). The doping of sulfur elements in S-GDY was quantitatively analyzed using XPS, and the results showed that the atomic percentage of sulfur was 4.39% (Additional file 1: Table S1). XPS can observe a weak peak of S2p (162 eV), indicating that sulfur has been successfully doped in GDY (Fig. 1D). The peak in the C 1 s XPS spectrum of S-GDY can be back integrated into four sub-peaks, including C=C (sp2) at 284.4 eV, C≡C (sp) at 285.0 eV, C–S at 286.3 eV, and C=S at 288.7 eV (Fig. 1E).Whereas for the refined spectrum of S 2p (Fig. 1F), three sub-peaks were assigned as (C)-SOx-(C) (168.4 eV), S 2p1/2 (163.1 eV), and S 2p3/2 (161.5 eV). The (C)-SOx-(C) peak might originate from SOx groups attached to the GDY network, the S 2p1/2 peak may be assigned to the substituted sulfur atom in the benzene ring of GDY, and the S 2p3/2 peak might be attributed to a thiol group attached to the GDY network [21]. The above results demonstrated that S-GDY nanosheets have been successfully prepared. In addition, the obtained S-GDY nanosheets were stable in water, PBS, RPMI-1640 medium, and RPMI-1640 medium+ 10% fetal calf serum (FBS), and only a slight increase in the size of S-GDY nanocomposite was observed within 72 h (Additional file 1: Fig. S1).

### Piezoelectric property of S-GDY

**Fig. 2:**
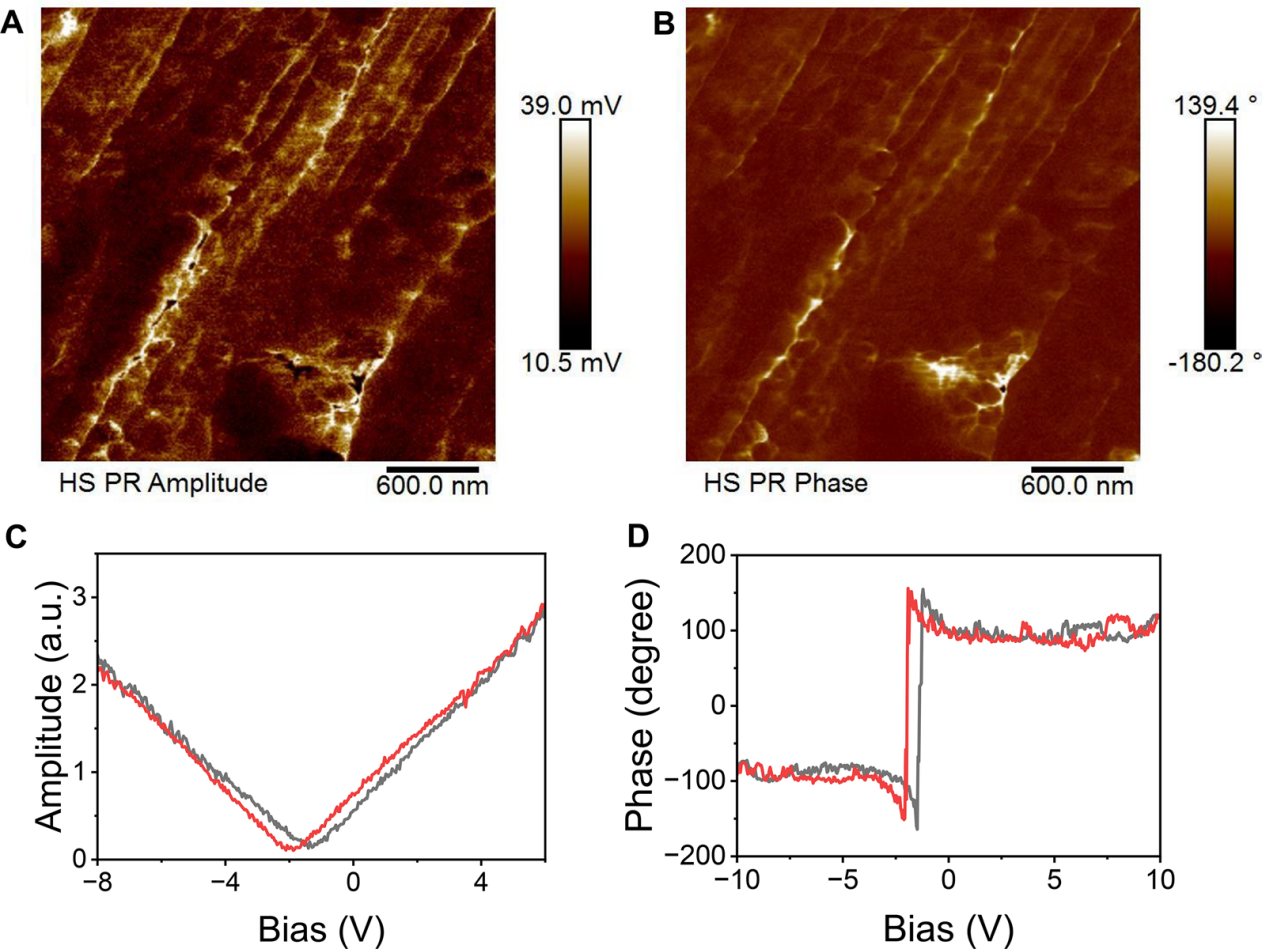
Piezoelectric properties of S-GDY. The amplitude map (**A**), phase diagram (**B**) amplitude butterfly loop (**C**) and phase lag loop of S-GDY (**D**). Note: The red and gray lines represent a cycle of voltage changes, with the red line from positive to negative pressure and the gray line from negative to positive pressure

The piezoelectric properties of S-GDY were confirmed using PFM. Piezoelectric surface oscillations were induced by applying an alternating voltage to the conductive cantilever tip S-GDY in contact mode. As shown in Fig. 2A, B, there is a clear contrast in the phase and amplitude of different regions in the PFM amplitude and phase maps, indicating the presence of several polarization directions. Figure 2C showed the butterfly loop due to S-GDY's electric field-induced polarization switching behavior, thereby causing the strain of electric field hysteresis. Explicit hysteresis loops indicate the presence of switchable polarization (Fig. 2D). Therefore, we determined that S-GDY has piezoelectric properties, which can lay the foundation for subsequent studies.

### Piezoelectric enhanced POD-like activity of S-GDY

**Fig. 3:**
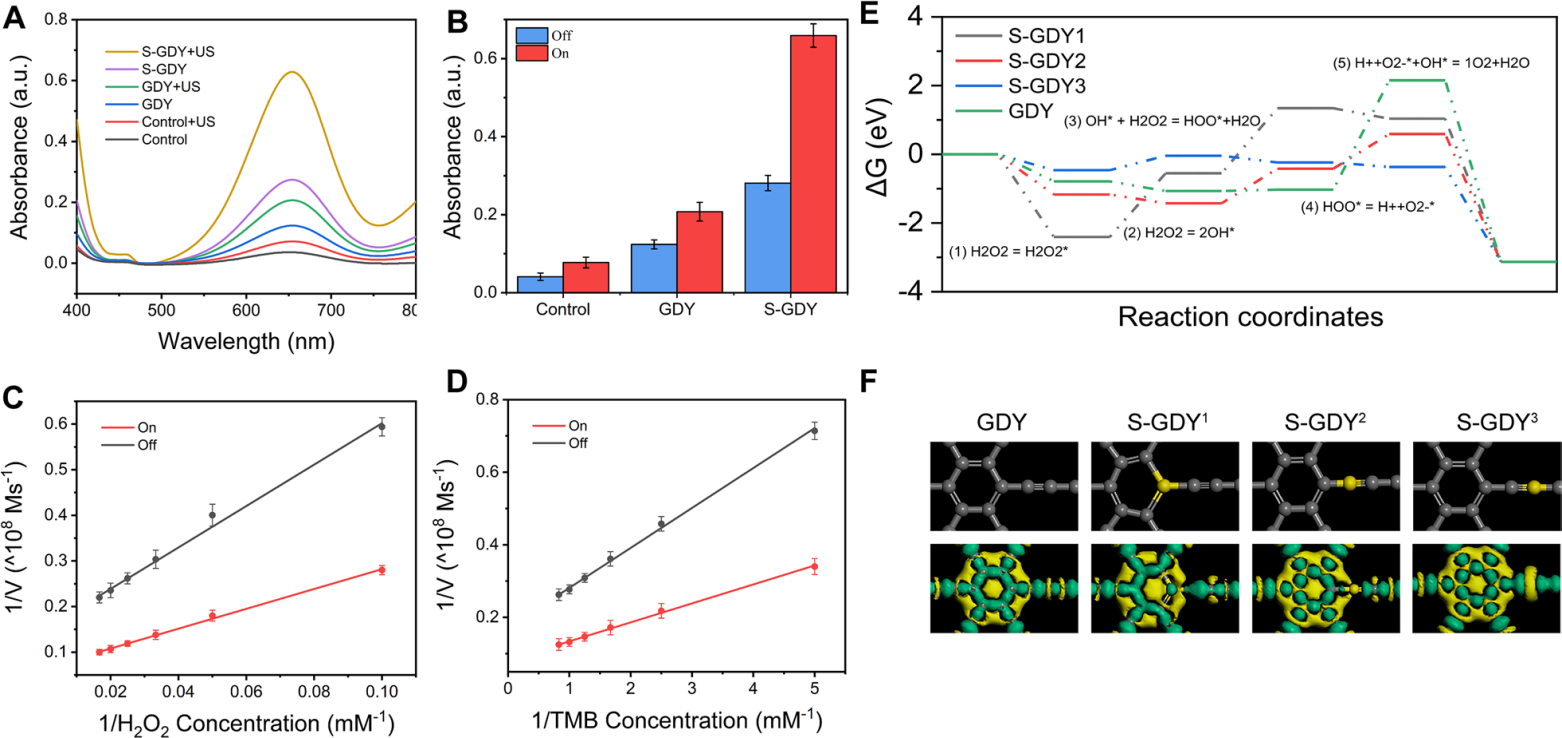
Piezoelectric enhanced peroxidase-like performance of S-GDY and corresponding mechanistic investigation. **A** TMB oxidation absorption spectra of different conditions with and without ultrasound, **B** Multiple piezoelectric enhancement under different conditions, **C** Comparison of kinetic rate constant of H_2_O_2_ with and without ultrasound, **D** Comparison of kinetic rate constant of TMB with and without ultrasound, **E** The potential energy profiles of GDY and S-GDY in the peroxidase-like reaction, **F** Theoretic models of GDY and S-GDY and their corresponding EDD spectra

The POD-like activity of S-GDY was evaluated using TMB. The ·OH produced by catalyzing H_2_O_2_ can oxidize TMB and show the characteristic peak at 652 nm. As depicted in Fig. 3A, compared with other groups, the S-GDY + US group showed the most substantial peak at 652 nm, which indicated that ultrasound irradiation could significantly enhance the POD-like activity of S-GDY. The piezoelectric enhancement of the POD-like activity of the S-GDY and control groups and the GDY group was quantified in Fig. 3B. Obviously, the synergy of piezoelectric properties and ultrasonic cavitation of S-GDY increases the absorbance of TMB to 234.5% of that of S-GDY.

Furthermore, the steady-state catalytic kinetics analysis was performed further to obtain the catalytic activity of S-GDY. The time-dependent curves of TMB were obtained by varying the concentrations of H_2_O_2_ and TMB to obtain Michaelis–Menten curves, the Michaelis–Menten constant (K_m_) and maximum velocity (V_max_) values were calculated by the Lineweaver–Burk equation (Additional file 1: Fig. S2, Additional file 1: Table S2). The V_max_ of H_2_O_2_ and TMB after ultrasonic irradiation were 11.49 × 10 − 8 M s^−1^ and 12.28.3 × 10 − 8 M s^−1^, respectively, which increased to 182.7% and 209.9% of those without ultrasonic irradiation (Fig. 3C, D). The promotion effect of ultrasound on S-GDY peroxidase. In addition, we tested the consumption capacity of S-GDY on GSH. The results showed that S-GDY had a significant GSH consumption capacity, which laid the foundation for the subsequent redox destruction of tumors and the induction of ferroptosis (Additional file 1: Fig. S3).

### DFT calculations

The full-path transition state reaction energies of S-GDY and H_2_O_2_ were investigated using DFT calculations. Gibbs free energy diagrams of POD-like reactions were built for the basal planes of multiple potential catalytically active sites to identify the active sites of each model and assess their POD-like activity (Fig. 3E). The largest Gibbs free energy change of GDY with any doping occurs on the basal plane of HOO* splitting into O^2−^ and H^+^ (ΔG = 2.15 eV). S-GDY has a lower maximum reaction barrier than GDY. Nevertheless, the Gibbs free energy changes during the formation of two HO* by homolytic cleavage of H_2_O_2_* were – 2.41 eV and – 1.17 eV, respectively, indicating sulfur becomes the active center for strong HO* adsorption [21]. S-GDY^1^ and S-GDY^2^ increased the number of catalytically active sites, thereby enhancing the catalytic activity. The lowest Gibbs free energy change of the rate-determining step occurred in the homolysis of hydrogen peroxide to HO* (ΔG = − 0. 04 eV) for S-GDY^3^.The potential barrier is very low. Therefore, S-GDY^3^ can effectively reduce the energy barrier and enhance POD-like activity compared with other analogues.

Figure 3F shows the electron density contrast (EDD) diagram that can observe the charge distribution in GDY and S-GDY. The green and blue areas represent the dissipation and accumulation of electrons, respectively. Electrons accumulate more easily at the sulfur atoms of S-GDY^1^ and S-GDY^2^ than GDY, making it easier to adsorb H_2_O_2_. The sulfur atom in S-GDY^3^ protrudes from the base, which may lower the energy barrier and place it at the active center of the reaction, making it easier to adsorb H_2_O_2_, causing H_2_O_2_ to occur homolysis evenly and producing reactive oxygen species.

### ROS Antitumor performance of S-GDY in vitro

Inspired by the excellent piezoelectric enhanced POD-like activity of S-GDY, the in vitro therapeutic performance was further performed against 4T1 cancer cells. The ability of S-GDY to be uptake by 4T1 cells into the cell is crucial for its therapeutic effect [38]. S-GDY was labeled with red fluorescent DiI dye and co-incubated with 4T1 cells. The results showed that with the prolongation of incubation time, the red fluorescence in 4T1 cells gradually increased, indicating that S-GDY has good cellular uptake ability (Additional file 1: Fig. S4).

**Fig. 4:**
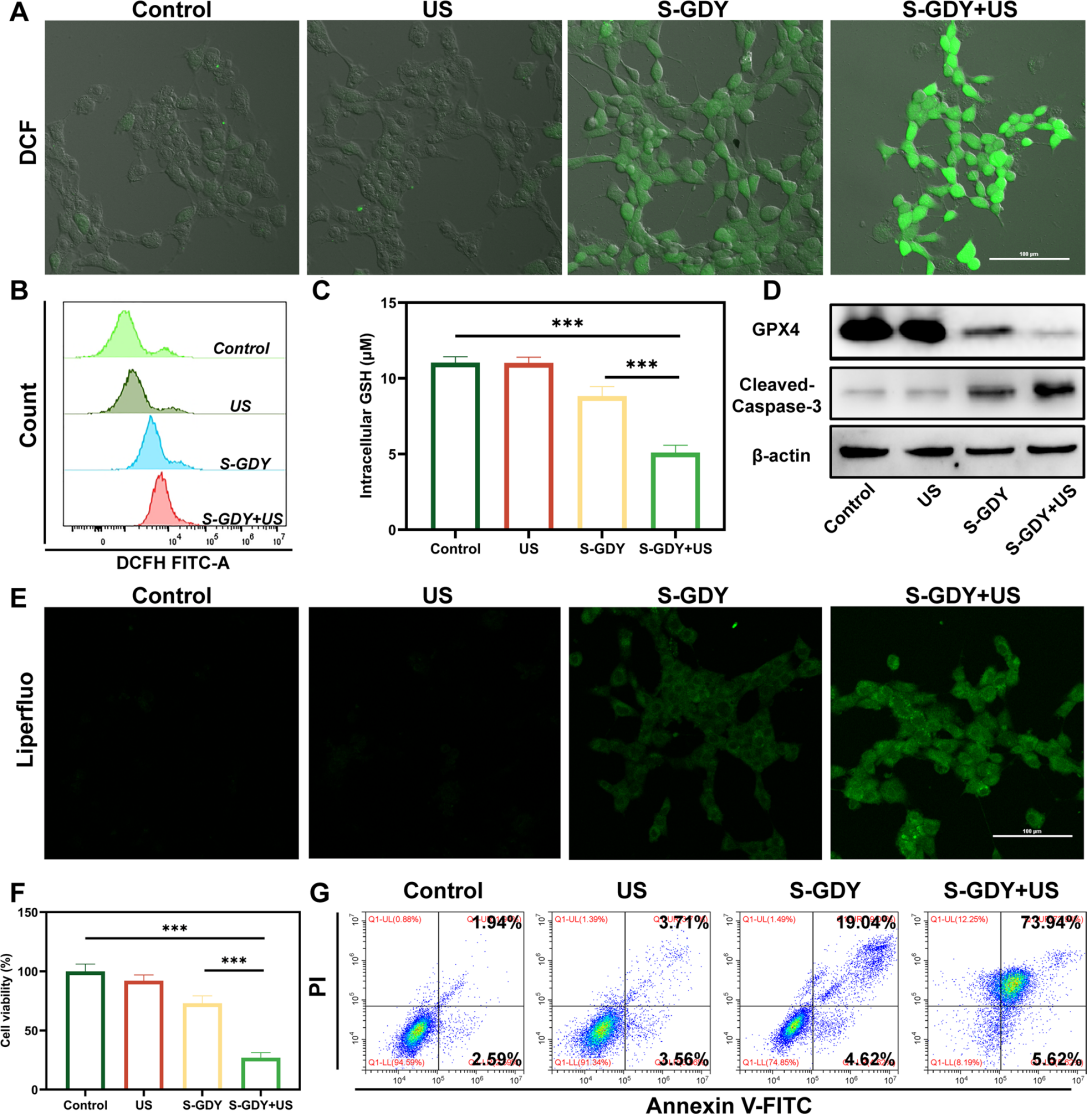
Antitumor performance of S-GDY in vitro. **A** CLSM images of intracellular total ROS production in 4T1 cells (scale bar: 100 µm), **B** Detection of intracellular total ROS production in 4T1 cells by flow cytometry, **C** The intracellular GSH levels after various treatments, **D** Western blot analysis on the expressions of GPX4 and Cleaved-Caspase-3, **F** Cell viability of 4T1 cells after different treatments, **G** The apoptosis of 4T1 cells with different treatments was detected by flow cytometry

Next, DCFH-DA was used to detect the generation of intracellular ·OH by CLSM. The fluorescence intensity of the S-GDY+ US group was stronger than that of the S-GDY group (Fig. 4A). The flow cytometry also showed the same experimental results (Fig. 4B). The above results indicate that US increases the ability of S-GDY nanosheets to produce ROS, which is beneficial for anti-tumor effects [29, 39]. Next, we detected the consumption level of GSH in 4T1 cells after various treatments at the cellular level (Fig. 4C). The results showed that the S-GDY+ US group showed more significant GSH depletion compared with the Control group and S-GDY group (*P* < 0.05), which may break the intracellular redox balance and lead to the therapeutic effect. Depletion of intracellular GSH will lead to the inactivation of GPX4.

Furthermore, we detected the expression level of GPX4 after various treatments by Western blot. We verified that the expression of GPX4 in the S-GDY + US group reduced (Fig. 4D). This leads to increased intracellular LPO levels. Therefore, we detected the accumulation of LPO in each group after treatment using the Liperfluo probe. The results showed that the S-GDY + US group showed the strongest green fluorescence, indicating the highest accumulation of LPO (Fig. 4E). The above results confirmed that the S-GDY + US group induced more 4T1 cells to undergo ferroptosis through GSH depletion, GPX4 inactivation, and LPO accumulation.

Caspase-3, as the main terminal cleaving enzyme in the process of apoptosis, encodes a 32 kDa protein, and Caspase-3 is cleaved and activated when apoptosis occurs, producing a large subunit of 17 kDa and a small subunit of 12 kDa [40–42]. Therefore, the occurrence of apoptosis is evaluated by detecting the amount of Cleaved-Caspase-3 (17 kDa) protein. The Western blot results showed that the S-GDY + US group will find more Cleaved-Caspase-3 expression, thereby inducing more apoptosis (Fig. 4D).

To quantitatively estimate the therapeutic effect of S-GDY on the 4T1 cell line, the CCK-8 protocol was used to assess in vitro cytotoxicity (Fig. 4F). The toxicity of pure ultrasonic irradiation to cells is negligible, and S-GDY has certain cytotoxicity. However, combining S-GDY and US, cell viability drops sharply to 27.0% (*P* < 0.05). Furthermore, to quantify the level of apoptosis in each group, the cells were stained with Annexin V-FITC and PI for flow cytometry detection after various treatments. In the S-GDY + US group, 79.56% of 4T1 cells were observed to undergo apoptosis, suggesting that ultrasound-mediated piezoelectric catalytic therapy has excellent therapeutic effects on 4T1 cells (Fig. 4G).

### The pharmacokinetics and metabolism of S-GDY in vivo

The half-life period of S-GDY nanosheets in the blood was determined to be 2.278 h by intravenous injection of DiR-labeled S-GDY (Additional file 1: Fig. S5). S-GDY nanosheets have a longer time in the blood circulation and can achieve tumor targeting in the body, followed by metabolism and excretion out of the body. Therefore, we next evaluated the tumor-targeting ability of S-GDY nanosheets, and the results showed that with the prolongation of injection time, the fluorescence in the tumor area gradually increased (Additional file 1: Fig. S6). In contrast, the fluorescence in the liver gradually decreased, indicating that they have good in vivo tumor targeting ability.

### Therapeutic effect of S -GDY in vivo

**Fig. 5:**
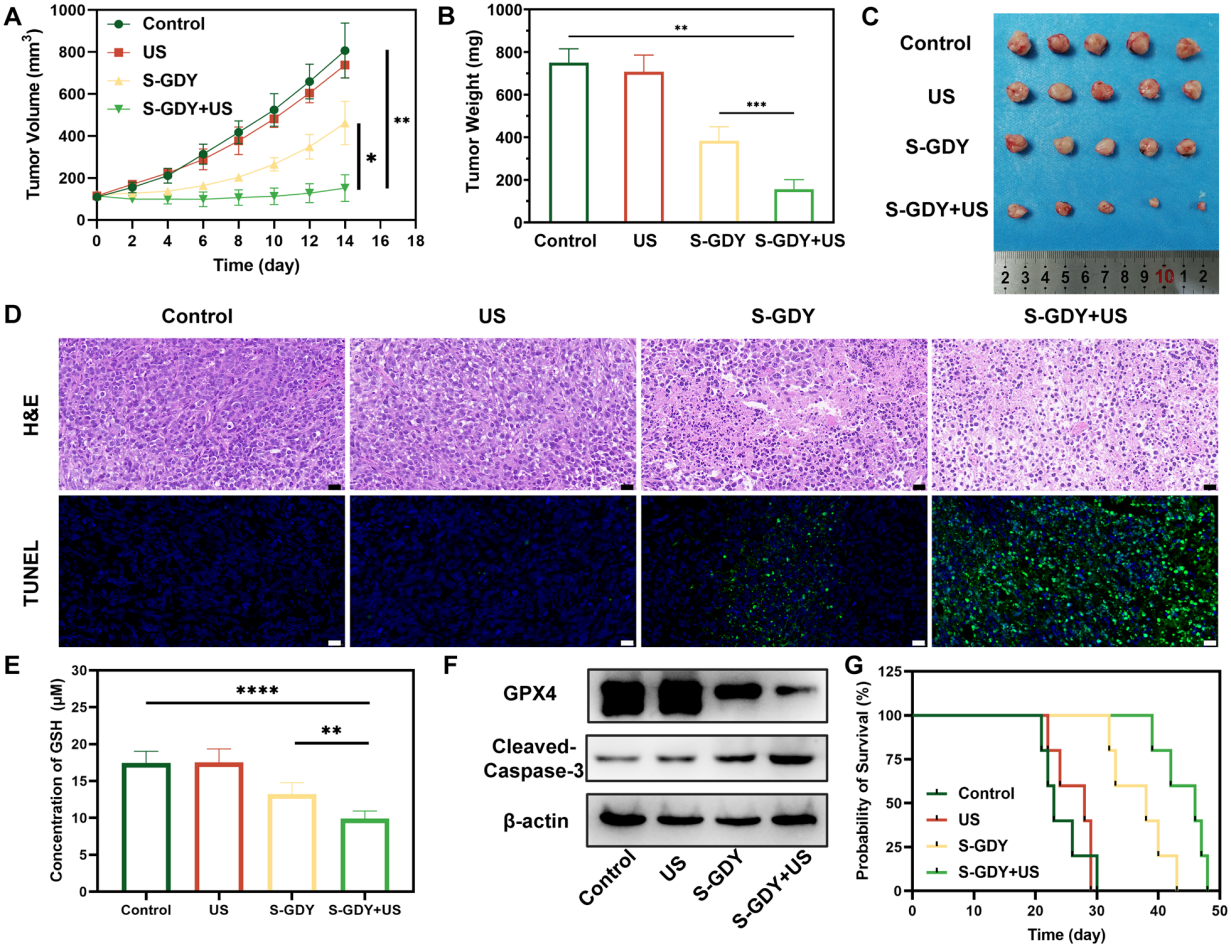
Antitumor performance of S-GDY in vivo. **A** Time-dependent tumor volume curves after different treatments (n = 5), **B** Tumor weight curves after different treatments (n = 5), **C** Photographs of tumors dissected from mice in four groups after various treatments (n = 5), **D** H&E and TUNEL staining of tumor regions after different treatments (scale bar: 20 µm), **E** Concentration of GSH in tumors (n = 5). **F** Western blot analysis on the expressions of GPX4 and Cleaved-Caspase-3 in tumors. **G** Survival time of 4T1 tumor-bearing mice (n = 5)

As evident in Fig. 5A, tumor volume was significantly reduced in the S-GDY + US group compared to the control groups, and the in vivo treatment effect of the S-GDY + US group is better than that of the S-GDY group (*P* < 0.05). After the treatment in vivo, the mice were sacrificed, and the tumor was weighed to obtain a result consistent with the tumor volume (Fig. 5B). The digital photos of tumor sites follow the progress of the tumor (Fig. 5C). H&E and TUNEL staining of tumor sections revealed that severe apoptosis occurred in the S-GDY + US group (Fig. 5D). The body weights of mice in four groups showed negligible fluctuations (Additional file 1: Fig. S7). No abnormal morphological changes were observed in major organs compared to the control group (Additional file 1: Fig. S8).

In addition, after treatment, the GSH levels in the tumors of each group were detected. As shown in Fig. 5E, it was found that the GSH levels in the S-GDY + US group were significantly reduced (*P* < 0.05). Compared with the control group, the expression of GPX4 protein in the S-GDY + US group was also significantly reduced, which also verified the occurrence of ferroptosis in this group from the in vivo level (Fig. 5F). Survival analysis showed that the S-GDY + US group had the highest survival rate and longest survival time in mice due to its excellent anti-tumor effect (Fig. 5G).

### Biological safety of S-GDY nanosheets

Compared with the control group, all S-GDY-administered groups showed little changes in routine blood tests and blood biochemical analysis (Additional file 1: Fig. S9). H&E staining of major organs (including heart, liver, spleen, lung, and kidney) from healthy Kunming mice showed negligible damage in vivo (Additional file 1: Fig. S10). Therefore, the innovative S-GDY nanosheets we constructed have good biological safety, which is beneficial for their clinical translation.

## Conclusions

In conclusion, this study reports a metal-free doped S-GDY nanozyme, which appears to be a highly efficient POD mimic and exhibits nonparallel piezoelectric responsiveness for enhanced enzyme mimicry activity. More importantly, the enhanced nanozyme induced 4T1 cell ferroptosis by promoting an imbalanced redox reaction due to GSH depletion and GPX4 inactivation. S-GDY exhibited enhanced nanozyme activity in vitro and in vivo that may directly trigger apoptosis-ferroptosis for effective tumor therapy. Altogether, this study is expected to provide new insights into the design of piezoelectric catalytic nanozymes and expand their application in the catalytic therapy of tumors.

### Supplementary Information

**Additional file 1: Table S1.** Atomic Composition of S-GDY. **Table S2.** Kinetic rate constant of H_2_O_2_ and TMB with and without ultrasound. **Fig. S1. **Particle size distribution of S-GDY at various time points in different solvents (n = 3). **Fig. S2. **Apparent steady-state kinetic study of S-GDY for (**A**) H_2_O_2_ and (**B**) TMB without ultrasound and (**C**) H_2_O_2_ and (**D**) TMB with ultrasound. **Fig. S3. **GSH consumption rates after different treatments. **Fig. S4.  **Cellular uptake of DiI-labeled S-GDY observed using CLSM (scale bar: 50 µm). **Fig. S5. **Circulating metabolism of DIR-labeled S-GDY in plasma. **Fig. S6. **In vivo and ex-vivo fluorescence images at different time intervals after intravenous injection of DIR-labeled S-GDY. **Fig. S7. **Time-dependent body weight curves after different treatments (n = 5). **Fig. S8. **H&E staining of major organs (heart, liver, spleen, lung, and kidney) after various treatments (scale bar: 50 µm). **Fig. S9. **Biochemical assay and hematology analysis of mice intravenously injected with S-GDY. The blood samples were collected post-injection at pre-determined time points (0, 1, 7, and 14 d). **Fig. S10. ** H&E staining of tissue sections from major organs after intravenously injected with S-GDY at pre-determined time points (0, 1, 7, and 14 d). The scale bar is 50 µm.

## Abbreviations

S-GDY: Sulfur-doped graphdiyne
US: Ultrasound
POD: Peroxidase
GDY: Graphdiyne
H_2_O_2_: Hydrogen peroxide
ROS: Reactive oxygen species
OH: Hydroxyl radicals
GSH: Glutathione
GPX4: Glutathione peroxidase 4
LPO: Lipid peroxidation
Na_2_S: Sodium sulfide
DTNB: 5,5′-Dithio-bis (2-nitrobenzoic acid)
TMB: 3,3′,5,5′-Tetramethylbenzidine
DCFH-DA: 2′-7′-Dichlorofluorescein diacetate
CCK-8: Cell Counting Kit-8
TEM: Transmission electron microscopy
PFM: Piezoelectric response force microscopy
XPS: X-ray photoelectron spectrometer
DFT: Density functional theory
CLSM: Confocal laser scanning microscopy
PBS: Phosphate buffered saline
FL: Fluorescence
K_m_: Michaelis–Menten constant
V_max_: Maximum velocity
EDD: Electron density contrast
TUNEL: TdT-dependent dUTP-biotin nick end labeling
H&E: Hematoxylin and eosin

## Declarations

### Availability of data and materials

All data generated or analyzed during this study are included in this manuscript and its additional files.
