# Supplementary material for: Piezoelectric enhanced sulfur doped graphdiyne nanozymes for synergistic ferroptosis–apoptosis anticancer therapy
Source: J Nanobiotechnology. 2023 Sep 2;21:311. doi: 10.1186/s12951-023-02059-y (PMC10474662; doi:10.1186/s12951-023-02059-y)
Supplement: Supplementary file 1 — Additional file 1: Table S1. Atomic Composition of S-GDY. Table S2. Kinetic rate constant of H2O2 and TMB with and without ultrasound. Fig. S1. Particle size distribution of S-GDY at various time points in different solvents (n = 3). Fig. S2. Apparent steady-state kinetic study of S-GDY for (A) H2O2 and (B) TMB without ultrasound and (C) H2O2 and (D) TMB with ultrasound. Fig. S3. GSH consumption rates after different treatments. Fig. S4. Cellular uptake of DiI-labeled S-GDY observed using CLSM (scale bar: 50 µm). Fig. S5. Circulating metabolism of DIR-labeled S-GDY in plasma. Fig. S6. In vivo and ex-vivo fluorescence images at different time intervals after intravenous injection of DIR-labeled S-GDY. Fig. S7. Time-dependent body weight curves after different treatments (n = 5). Fig. S8. H&E staining of major organs (heart, liver, spleen, lung, and kidney) after various treatments (scale bar: 50 µm). Fig. S9. Biochemical assay and hematology analysis of mice intravenously injected with S-GDY. The blood samples were collected post-injection at pre-determined time points (0, 1, 7, and 14 d). Fig. S10. H&E staining of tissue sections from major organs after intravenously injected with S-GDY at pre-determined time points (0, 1, 7, and 14 d). The scale bar is 50 µm. [file 12951_2023_2059_MOESM1_ESM.docx]

**Additional file 1**

**Piezoelectric enhanced sulfur doped graphdiyne nanozymes for synergistic ferroptosis -apoptosis anticancer therapy**

Jianxin Wang^1^, Yinzhu Chu^1^, Zhiyu Zhao^1^, Cong Zhang^1^, Qi Chen^1^, Haitao Ran^2^, Yang Cao^2, *^, Changjun Wu^1, *^

1 Department of Ultrasound, The First Affiliated Hospital of Harbin Medical University, Harbin 150001, China

2 Chongqing Key Laboratory of Ultrasound Molecular Imaging, Institute of Ultrasound Imaging, Second Affiliated Hospital, State Key Laboratory of Ultrasound in Medicine and Engineering, Chongqing Medical University, Chongqing 400010, China.

***Corresponding authors.**

E-mail addresses: bccjw@sohu.com (C. Wu), yangcao@cqmu.edu.cn (Y. Cao).

Keywords: Graphdiyne; Nanozymes; Ferroptosis; Ultrasound; Piezoelectric catalytic Therapy

**Table S1.** Atomic Composition of S-GDY.

| Name | Peak BE | FWHM eV | Atomic % |
| --- | --- | --- | --- |
| C 1s | 284.80 | 1.38 | 80.89 |
| O 1s | 532.53 | 2.7 | 14.72 |
| S 2p | 161.47 | 3.06 | 4.39 |





**Fig. S1.**Particle size distribution of S-GDY at various time points in different solvents (n = 3).

**
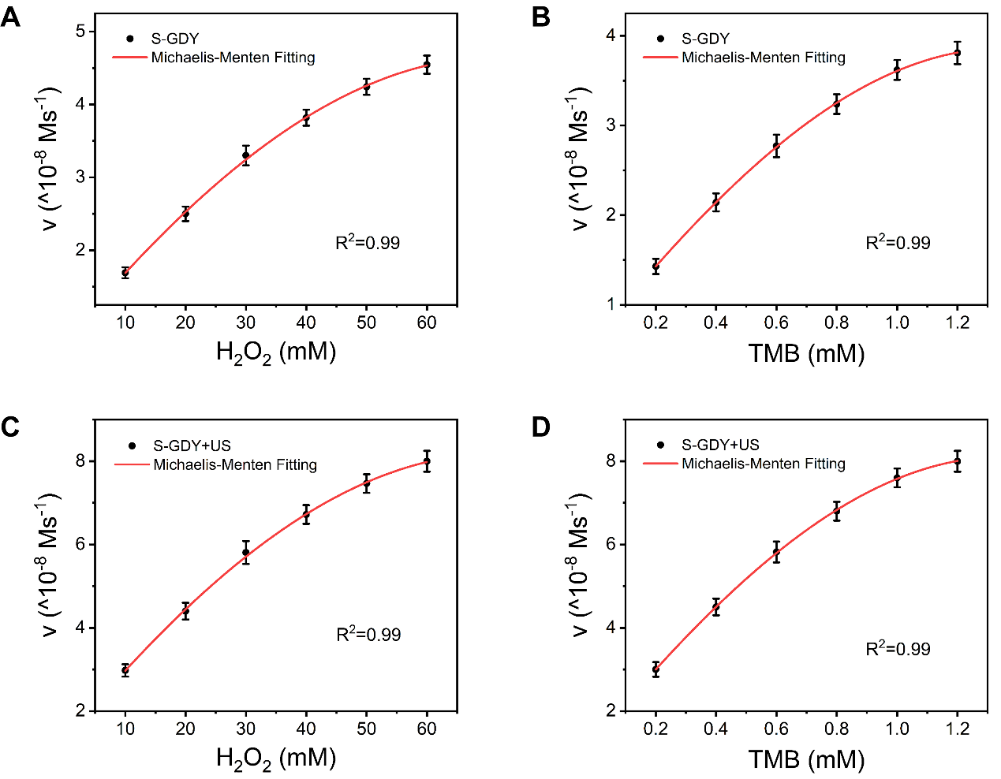
**

**Fig. S2.**Apparent steady-state kinetic study of S-GDY for (A) H_2_O_2_ and (B) TMB without ultrasound and (C) H_2_O_2_ and (D) TMB with ultrasound.

**Table S2.** Kinetic rate constant of H_2_O_2_ and TMB with and without ultrasound.

| **Materials** | **Ultrasound** | **H_2_O_2_** | | **TMB** | |
| --- | --- | --- | --- | --- | --- |
|  |  | **Km (mM)** | **Vm (M s^-1^)** | **Km (mM)** | **Vm (M s^-1^)** |
| S-GDY | On | 26.51 | 11.49×10^-8^ | 0.64 | 12.26×10^-8^ |
|  | Off | 28.58 | 6.29×10^-8^ | 0.65 | 5.84×10^-8^ |





**Fig. S3.**GSH consumption rates after different treatments.


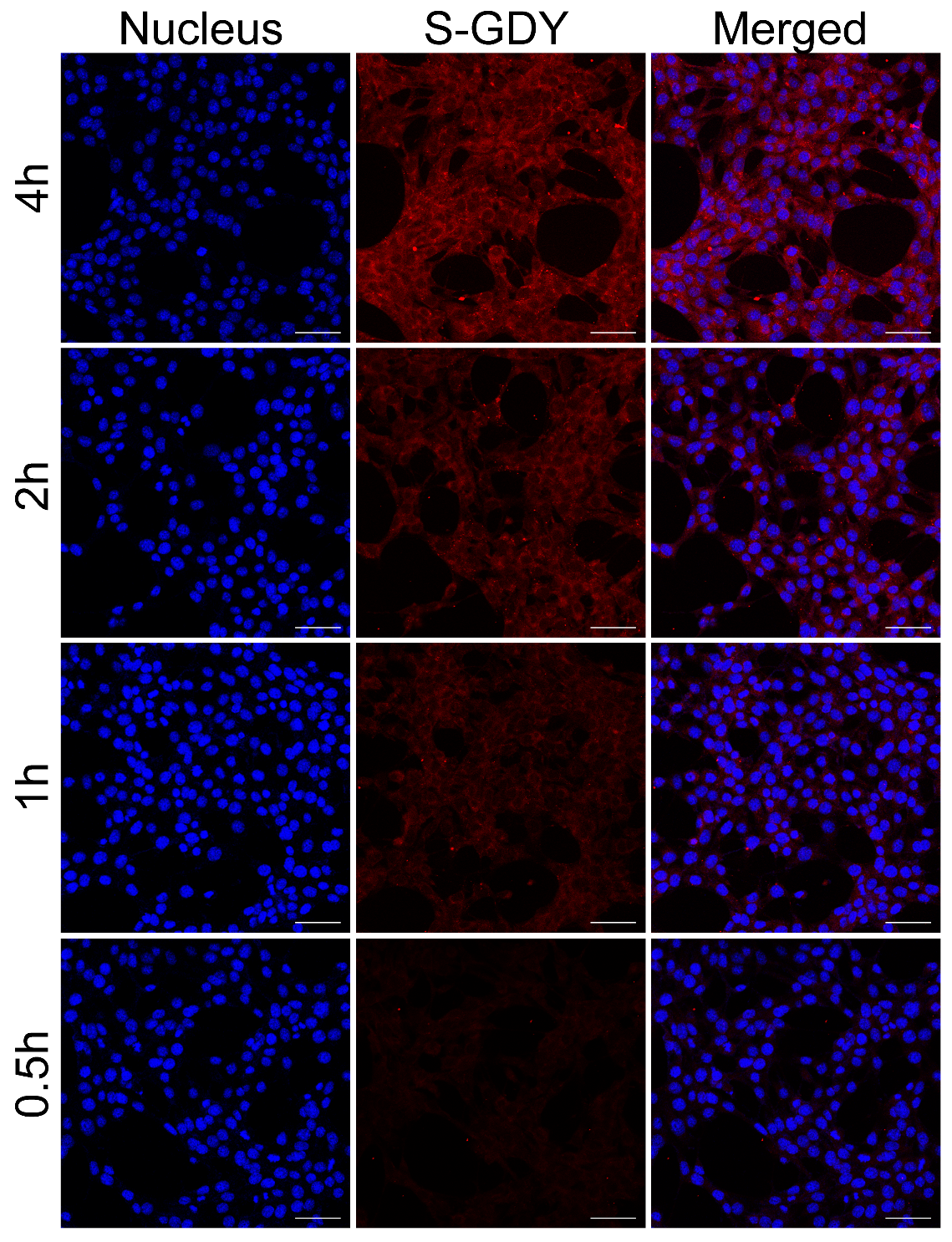


**Fig. S4.** Cellular uptake of DiI- labeled S-GDY observed using CLSM (scale bar: 50 µm).





**Fig. S5.**Circulating metabolism of DIR-labeled S-GDY in plasma.


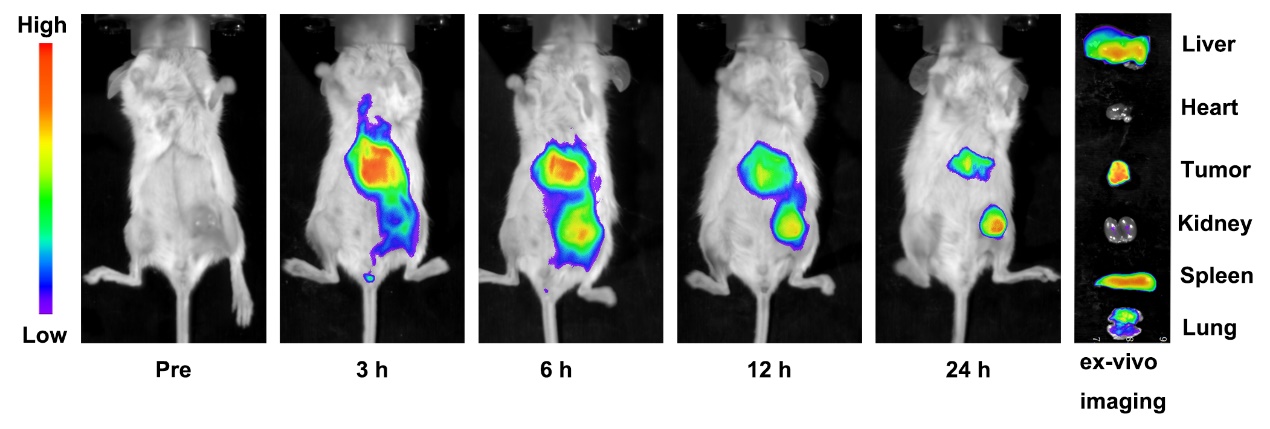


**Fig. S6.**In vivo and ex-vivo fluorescence images at different time intervals after intravenous injection of DIR-labeled S-GDY.


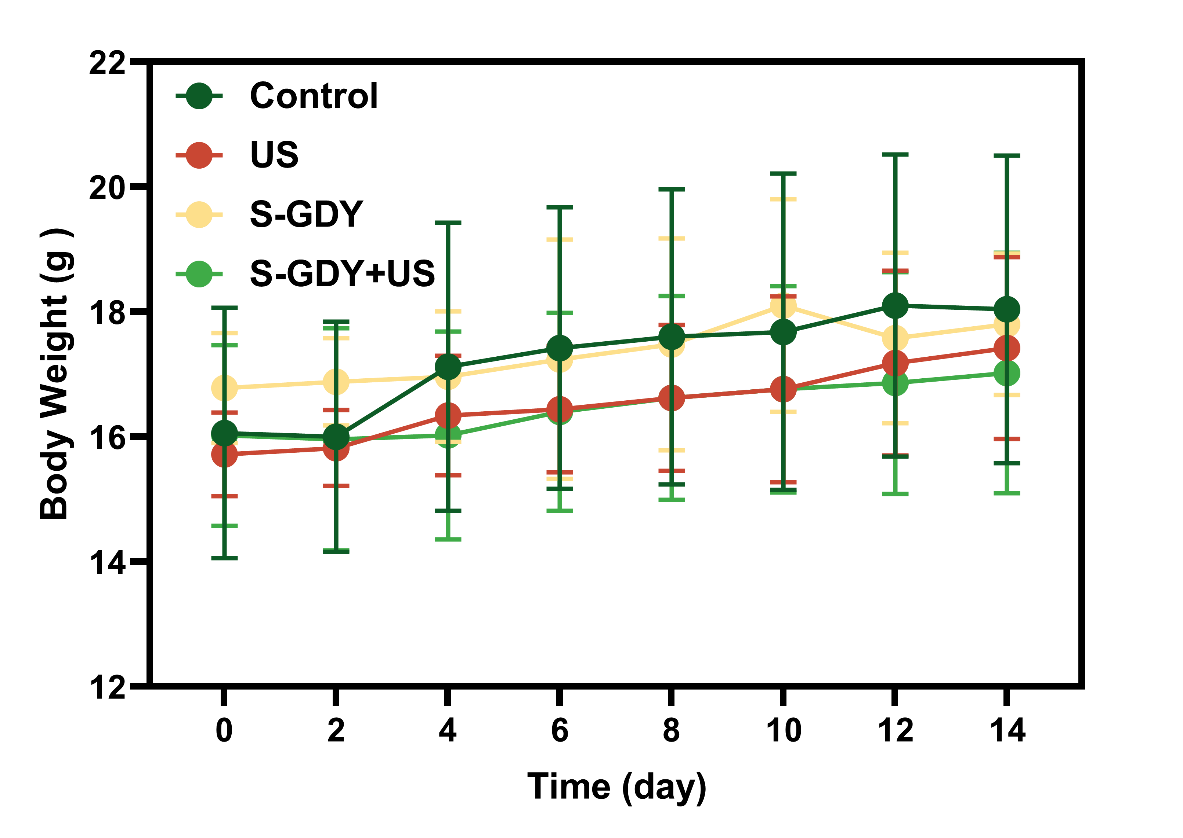


**Fig. S7.**Time-dependent body weight curves after different treatments (n = 5).


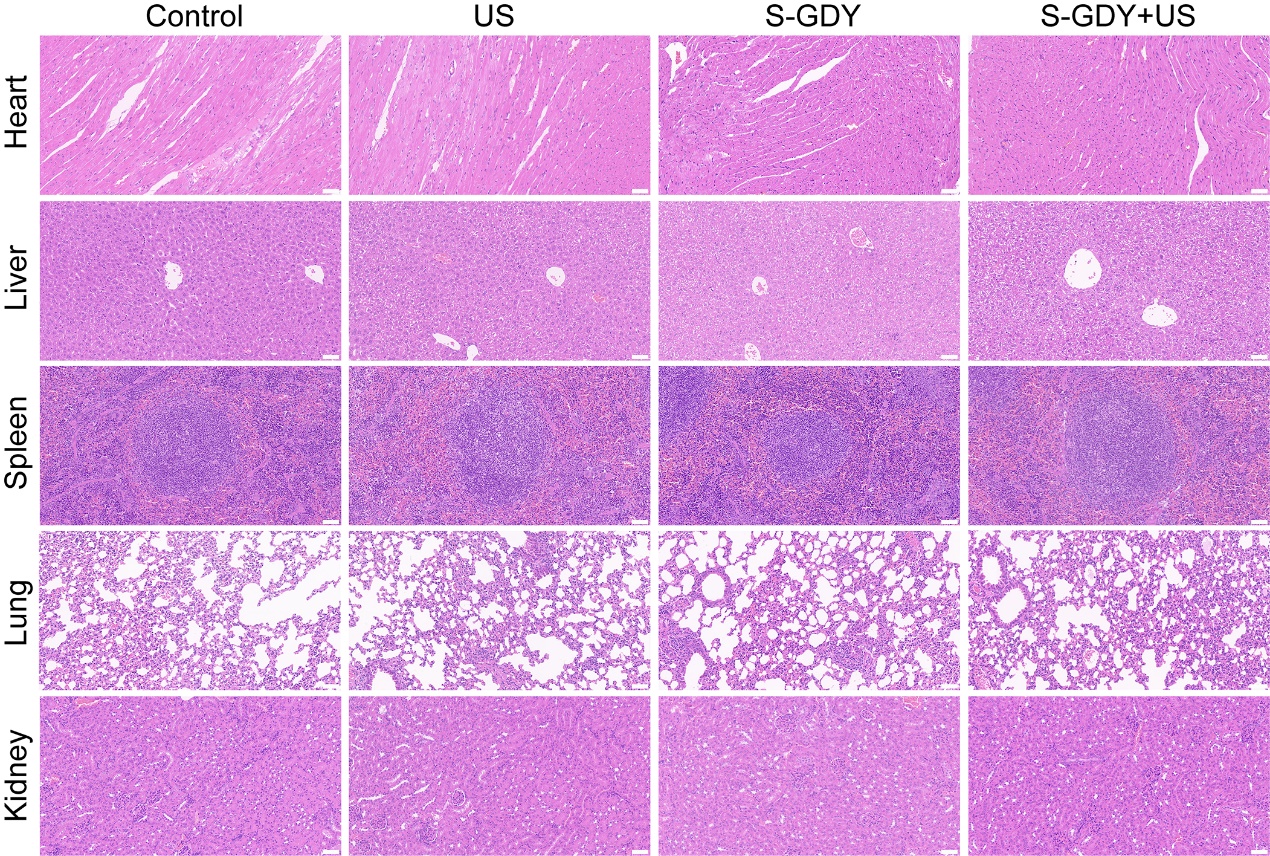


**Fig. S8.**H&E staining of major organs (heart, liver, spleen, lung, and kidney) after various treatments (scale bar: 50 µm).


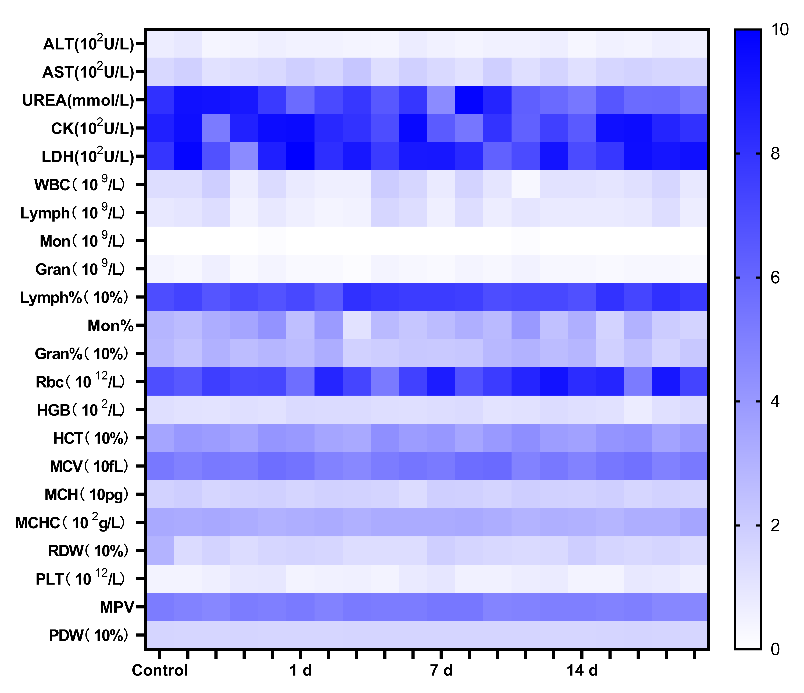


**Fig. S9.** Biochemical assay and hematology analysis of mice intravenously injected with S-GDY. The blood samples were collected post-injection at pre-determined time points (0, 1, 7, and 14 d).


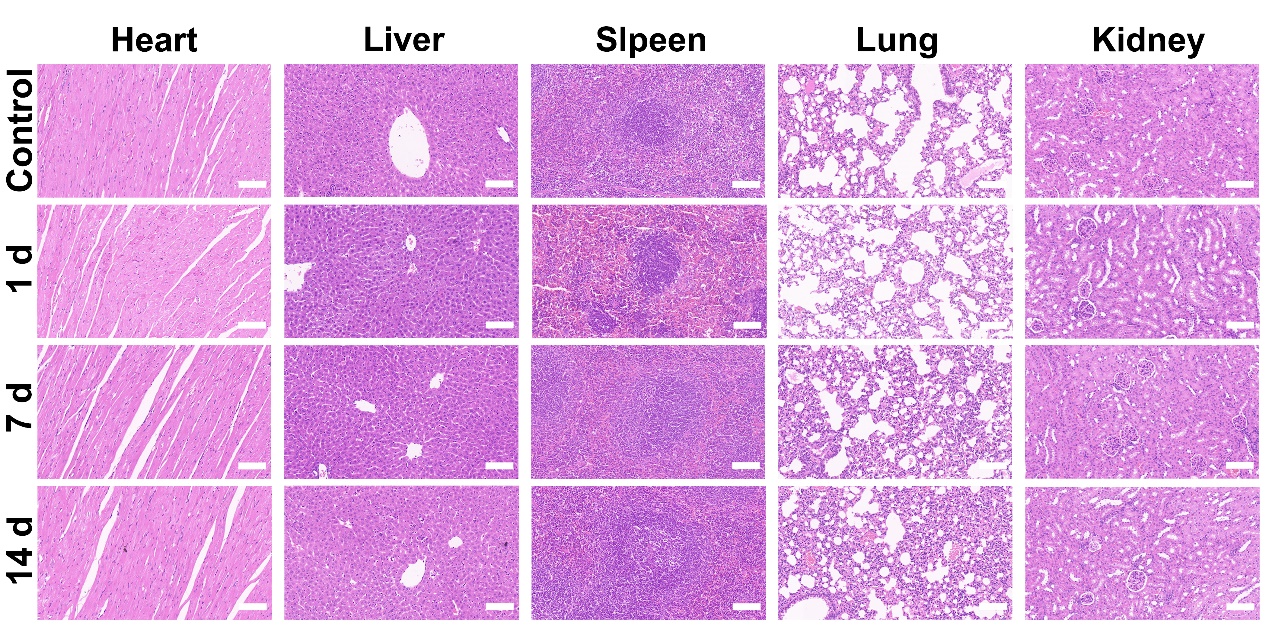


**Fig. S10.** H&E staining of tissue sections from major organs after intravenously injected with S-GDY at pre-determined time points (0, 1, 7, and 14 d). The scale bar is 50 µm.
